# The Adversarial Robust and Generalizable Stereo Matching for Infrared Binocular Based on Deep Learning

**DOI:** 10.3390/jimaging10110264

**Published:** 2024-10-22

**Authors:** Bowen Liu, Jiawei Ji, Cancan Tao, Jujiu Li, Yingxun Wang

**Affiliations:** 1 School of Automation Science and Electrical Engineering, Beihang University, 37 Xueyuan Road, Haidian District, Beijing 100191, China; jjw1999@buaa.edu.cn (J.J.);; 2 Faculty of Arts, Business, Law, and Economics, University of Adelaide, North Terrace, Adelaide, SA 5005, Australia; lijujiusally@foxmail.com

**Keywords:** deep learning, stereo matching, robust, generalization, infrared binocular, patch attack

## Abstract

Despite the considerable success of deep learning methods in stereo matching for binocular images, the generalizability and robustness of these algorithms, particularly under challenging conditions such as occlusions or degraded infrared textures, remain uncertain. This paper presents a novel deep-learning-based depth optimization method that obviates the need for large infrared image datasets and adapts seamlessly to any specific infrared camera. Moreover, this adaptability extends to standard binocular images, allowing the method to work effectively on both infrared and visible light stereo images. We further investigate the role of infrared textures in a deep learning framework, demonstrating their continued utility for stereo matching even in complex lighting environments. To compute the matching cost volume, we apply the multi-scale census transform to the input stereo images. A stacked sand leak subnetwork is subsequently employed to address the matching task. Our approach substantially improves adversarial robustness while maintaining accuracy on comparison with state-of-the-art methods which decrease nearly a half in EPE for quantitative results on widely used autonomous driving datasets. Furthermore, the proposed method exhibits superior generalization capabilities, transitioning from simulated datasets to real-world datasets without the need for fine-tuning.

## 1. Introduction

Stereo matching has been a fundamental and enduring challenge in the field of computer vision, extensively studied over several decades. It holds significant importance in a wide range of applications, particularly in autonomous driving and unmanned aerial vehicle (UAV) navigation.

Recently, deep learning-based approaches have demonstrated remarkable effectiveness for infrared binocular stereo matching algorithms, yielding more accurate and robust depth predictions. Deep neural networks (DNNs) have, much like in other areas of computer vision, driven significant progress in stereo matching [1,2,3,4]. However, while their widespread adoption has greatly enhanced performance, it has also uncovered new vulnerabilities. Advanced techniques have shown that even slight perturbations to the inputs can mislead these networks, leading to drastically altered predictions. This adversarial susceptibility has escalated into a critical issue, especially for safety-critical applications, sparking an intense wave of research focused on increasingly sophisticated attacks that frequently outpace the development of defensive strategies [5,6,7,8,9,10]. Balancing accuracy and adversarial vulnerability have become a formidable challenge within cutting-edge computer vision systems [11,12,13]. Addressing this challenge is of paramount importance to ensure the reliability and safety of modern vision systems. Therefore, developing robust stereo matching algorithms that can withstand adversarial attacks is essential for advancing the field and enabling the safe deployment of autonomous technologies.

Given the critical role of stereo matching methods in autonomous driving and navigation systems, addressing their adversarial vulnerabilities is imperative for ensuring safety and reliability in real-world applications. Previous research [14] has demonstrated that independent attacks on stereo images can introduce perturbations that disrupt the color consistency of projections from the same physical point. While these perturbations are significant in controlled experiments, they often lack practical relevance due to their unrealistic nature. To rigorously evaluate the real-world vulnerabilities of stereo matching techniques, we introduce the stereo constrained projected gradient descent (PGD) attack. This approach enforces photometric consistency between corresponding pixels, providing a more realistic framework for assessing the robustness of these algorithms. Our findings reveal that even when color integrity is preserved, state-of-the-art stereo matching models remain highly susceptible to adversarial attacks. This underscores a critical vulnerability in existing systems and highlights the urgent need for developing more resilient approaches to safeguard against such threats, thereby ensuring dependable performance in safety-critical applications like autonomous navigation.

Conventional methods to mitigate adversarial attacks predominantly rely on adversarial training [6]. While this strategy offers certain benefits, it also presents challenges, including performance degradation, extended training durations, and a propensity to overfit to specific attacks and datasets. To overcome these limitations, we advocate for the integration of domain-specific knowledge to enhance the inherent robustness of neural networks. The strong photometric consistency inherent in stereo images presents a unique opportunity to fortify defenses against adversarial attacks through intelligent network design.

In stereoscopic images, corresponding pixels from the same physical point in non-occluded regions generally exhibit similar colors—a property that holds true for infrared stereoscopic images when disregarding infrared texture. Leveraging DNN features for matching inadvertently increases the matching cost for features associated with attacked physical points. To address this issue, we propose an innovative approach that eschews reliance on DNN-derived features for matching, opting instead for handcrafted features that preserve minimal color differences between true correspondences. Specifically, we employ local binary patterns, such as the census transform [15,16], which compare each pixel’s intensity with that of its neighbors. This strategy enhances robustness by making the matching cost resistant to manipulation, thereby mitigating the impact of adversarial attacks.

While DNN features excel in providing high-level semantic information essential for estimating occluded and textureless regions, we restrict their usage to merely contextualizing the reference image. This context-enriched non-parametric cost quantity is processed by the head subnet, which acts as a learnable optimizer, to achieve the optimal matching result. In essence, we redirect stereo matching as a cost aggregation and optimization problem over a non-parametric cost quantity.

Experimental results underscore that this transparent approach significantly enhances adversarial robustness while preserving high accuracy. This method leverages the inherent strengths of both handcrafted and neural network features, offering a resilient and effective solution to adversarial challenges in stereo matching.

Beyond adversarial vulnerabilities, cross-domain generalization remains a pivotal challenge in stereo matching with great importance in application of practice. Due to the substantial costs involved in gathering accurate ground-truth data and the significant data demands of deep neural networks (DNNs), stereo matching algorithms frequently rely on simulation-to-reality (Sim2Real) training frameworks. Such methodologies, however, may lead DNNs to adopt shortcut strategies that are overly dependent on the idiosyncrasies of the training datasets [17].

By removing the DNN feature backbone from the matching process, we can repurpose the DNN as a more generalized cost volume optimizer. This modification reduces the likelihood of shortcut learning within the feature space, thereby improving cross-domain performance, especially in cases where fine-tuning is not applied. Our experiments demonstrate the effectiveness of this approach, showcasing improved generalizability from the SceneFlow dataset [18] to the KITTI benchmark [19] without the need for fine-tuning. The result compared with the mainstream reveals the adversarial attack robustness as well as virtual and reality cross-generalization of our method which has practical significance for autonomous driving and UAV navigation.

## 2. Materials and Our Contributions

Although infrared binocular stereo matching adds infrared texture, it is still binocular stereo matching in essence. Since ref. [20] introduced the first deep learning approach for stereo matching, significant progress has been made in this field. The development of DispNet by [18] as the first DNN-based end-to-end trainable method is a remarkable milestone. In addition to this, we also constructed SceneFlow, a large-scale synthetic dataset containing around 40,000 images.

In GCNet, the end-to-end methodology in [21] is enhanced through several significant innovations. These include integrating features at the cost volume stage, utilizing 3D convolutional layers for cost aggregation, and adopting a soft argmin operator for estimating disparities. These advancements have set a precedent, influencing subsequent methods that typically incorporate these design strategies and utilize the SceneFlow dataset for pretraining [19].

Significant enhancements in the cost aggregation phase have been reported in the literature [22,23,24]. Chang et al. introduced the application of the spatial pyramid pooling (SPP) module for feature extraction, alongside stacked hourglass structures for cost aggregation [25]. Furthermore, advances have been made in accelerating stereo matching by utilizing highly optimized, hand-crafted features such as census transformations and sum of absolute differences [26]. Both theoretical advancements have been integrated into our methodology.

Recent advancements have focused on enhancing stereo matching algorithms by reducing reliance on extensive 3D convolutional layers, with [27,28] proposing cost spatial propagation techniques. Further, to optimize performance, ref. [29] employed neural architecture search (NAS) technology to autonomously determine the most effective architecture for each phase of processing. These methodologies are currently at the forefront of the KITTI 2015 benchmark, representing the cutting edge in stereo matching technology [19].

Considering the analogous nature of infrared binocular stereo matching and conventional binocular stereo matching, the attack methodologies are expected to exhibit considerable similarity. With unrestricted access to DNNs pretrained on clean images, white-box targeted attacks have become powerful instruments for exposing the vulnerabilities of these networks. Many white-box attack strategies focus on objective functions constrained by norm-balls [30,31,32,33]. The integration of momentum in the MIFGSM method [33] and the use of $l_{p}$ gradient projection in the PGD method [6] have significantly improved their effectiveness in generating adversarial examples.

In actual navigation applications, physically realizable attacks have been explored across numerous tasks [34,35,36,37,38], with the notable exception of stereo matching. DNN-based stereo matching methods are vulnerable to unconstrained adversarial attacks against a single image [14], which has been proved in theory, but these attacks cannot maintain photometric consistency, thus violating the basic physical properties of binocular vision, making them impractical. Consequently, unconstrained attacks are incapable of creating adversarial patches that can deceive stereo systems. To address this, we propose a stereo constrained PGD attack specifically designed to assess the adversarial robustness of preserving photometric consistency in more realistic scenarios. While ref. [35] investigated adversarial attacks in optical flow—an area inherently less challenging than stereo matching due to the simpler nature of its problems—our study builds on Ranjan’s findings to illuminate potential avenues for developing more resilient optical flow networks.

Adversarial training remains the most widely used method for improving adversarial robustness [6,39]. However, it suffers from significant drawbacks, including diminished accuracy, extended training times, and overfitting to particular datasets and attacks. Although adversarial training can be applied across various DNNs, our approach enhances intrinsic robustness by exploiting the photometric consistency inherent in stereo matching, thus avoiding these limitations. Moreover, it can be combined with adversarial training to achieve even higher levels of robustness.

This paper describes three principal contributions to the field of infrared binocular stereo matching:

1. It demonstrates a deep integrative learning paradigm by re-evaluating the end-to-end DNN feature backbone in stereo matching, illustrating that the stereo matching algorithm can effectively adapt to infrared binocular images with a more accurate result.
2. It presents a novel stereo matching design that exhibits significantly improved adversarial robustness and cross-domain (Sim2Real) generalizability, even without the need for fine-tuning.
3. It introduces a stereo constrained projection gradient descent (PGD) attack method, which is proposed to maintain the consistency of photometry by design, revealing the core vulnerabilities of the accurate DNN-based stereo matching methods.

## 3. Approach

In this section, we describe the proposed method and the three-dimensional constrained PGD attack technique, which aims to evaluate the vulnerability of the DNN-based three-dimensional matching algorithm.

### 3.1. Our Proposed Method

**Figure 1:**
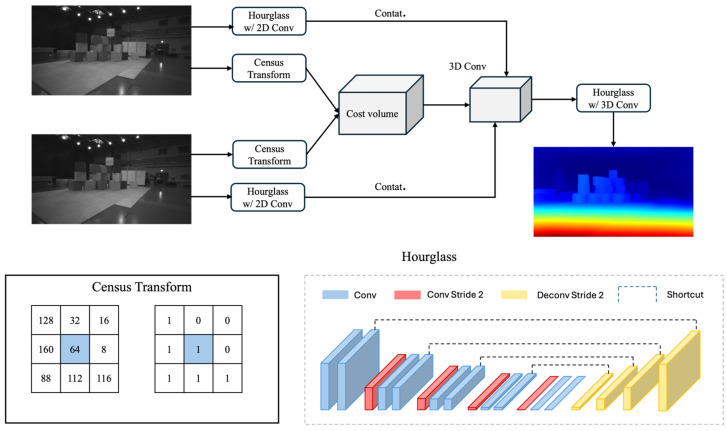
Workflow.

As illustrated in Figure 1, the proposed workflow comprises three primary components.

#### 3.1.1. Computing the Cost Volume Using Multi-Scale Census Transform

Contemporary stereo matching techniques predominantly utilize DNN-based features to formulate 4D cost volumes. While these features enhance the distinctiveness of pixel characteristics, their inherent properties render them susceptible to adversarial attacks. In contrast, traditional methods typically employ straightforward window-based similarity functions for initial cost estimation, subsequently integrating local cost data through optimization or aggregation phases [40]. Given this context, we advocate for the incorporation of hand-crafted feature descriptors and similarity functions during the initial phase. These elements are less prone to adversarial perturbations and permit the subsequent use of DNNs for the effective integration of local cost information.

Our objective was to maintain the integrity of feature descriptors under local intensity variations, prompting our adoption of the census transform. This traditional feature descriptor effectively addresses issues arising from radiometric disparities due to varying exposure times or non-Lambertian surfaces. Prior studies have established the census transform as one of the most resilient and adaptable cost functions, particularly when implemented alongside global or semi-global optimization techniques [15,16].

Unlike traditional color binocular images, infrared images are typically monochromatic, appearing in shades of black and white. To compute the census transform, we utilize the grayscale raw intensity values. Within a local window patch W centered at a pixel u in Λ, the census transform calculates the local binary pattern by comparing each neighboring pixel v in W with u. It assigns a value of 1 if $I\left(v\right)\geq I\left(u\right)$, and 0 otherwise. The Hamming distance, which measures the discrepancy between two binary strings, is then employed to determine the cost between corresponding patches.

In contrast to traditional semi-global or global stereo matching methods, where costs are scalar values, our approach leverages the adaptability of deep neural networks (DNNs) to implement a multi-scale census transform. Like the workflow census transform (CT) on the left of Figure 1, the value of the pixel given by the picture, whatever it may be (e.g., color picture, infrared image, SceneFlow, KITTI 2015), will be defined during the interval from 0~255. Under the blanket of CT, all the values around the center pixel will contrast with it and will be 1 if the value is bigger than the center pixel, and vice versa. This method can enhance the attacks as well as the cross-domain generalization because it focuses on the relative relationship between pixels rather than the absolute intensity value which requires the attacker to perform precise perturbations on a large number of pixels to significantly change features to make the attack. Although the color information of the image may be lost, by using features generated by CT in the matching process, the reliance on deep features is reduced. It does not require complex parameter tuning or extensive training processes, and this simplicity reduces reliance on specific data distributions. Since the information it utilizes typically remains consistent across different scenarios, the method more readily generalizes to diverse datasets, thereby reducing the risk of being attacked and increasing the generalization to some extent.

This also enhances the incorporation of contextual information across various scales. Specifically, we employ local windows with sizes ranging from $k_{1}$ to $k_{2}$ (e.g., $k_{1}=3$, $k_{2}=11$ in our experiments), resulting in $K=k_{2}-k_{1}+1$ (e.g., 9) costs associated with each matching candidate pair. To standardize costs across scales, the Hamming distance is normalized by the number of pixels within each local window. For an input stereo image pair $I_{L}$ and $I_{R}$ with spatial dimensions H times W, and assuming the maximum disparity level δ, the initial cost volume is a 4D tensor of size H×W×δ×K. To reduce computational costs, we employ 3D convolutions to downscale the cost volume to $\frac{1}{3}H\times \frac{1}{3}W\times \frac{1}{3}\delta \times C$, where C=32 is the number of channels, as is commonly seen in prior work.

#### 3.1.2. Contextualizing the Cost Volume and Aggregating the Cost

While robust to adversarial attacks, the census transform-based cost volume alone is insufficient to effectively handle occlusions and more complex semantic information, such as transparent objects and specular reflections. To address these challenges, we introduce a two-stack hourglass module with 2D convolutions to extract contextual information from the left reference image. This process yields a $\frac{1}{3}H\times \frac{1}{3}W\times C$ feature map, which is then unsqueezed along the second dimension (i.e., replicated $\frac{1}{3}\delta$ times) to match the size of the downscaled cost volume. These two components are subsequently concatenated along the penultimate dimension.

The contextualized cost volume is then processed through a three-stack hourglass module with 3D convolutions during the cost aggregation stage.

#### 3.1.3. Disparity Map Prediction and the Loss Function

To predict the final disparity map $D\left(u\right)$, for all u in Λ, the output from each stack within the hourglass module of the cost aggregation process is initially upsampled to the original dimensions H×W×l, represented as $D_{s}\left(x,y,d\right)$ where s denotes the stack index. Following the approach described in [21], the predicted disparity map $D_{s}\left(x,y\right)$ is then computed by:

(1) $$D_{s}\left(x,y\right)=\sum_{d=1}^{l}d\times Softmax\left(D_{s}\left(x,y,d\right)\right)$$

where Softmax is applied along the last dimension in $D_{s}\left(x,y,d\right)$.

During training, we utilize the smooth $L_{1}$ loss due to its robustness at disparity discontinuities and reduced sensitivity to outliers [22,27]. Given the ground-truth disparity map $D^{*}\left(u\right)$, the loss is defined as:

(2) $$Loss(\Theta ;D^{*})=\sum_{s=1}^{S}\beta_{s}\cdot \frac{1}{\left|\Lambda \right|}\sum_{u\in \Lambda }{Smooth}_{L_{1}}\left(D_{s}\left(u\right)-D^{*}\left(u\right)\right)$$

where Θ encompasses all parameters in the model, $\beta_{s}$ denotes the weight for the output from each stack s (e.g., 0.5, 0.7, and 1 are used for the 3-stack hourglass module in our experiments), $u=\left(x,y\right)$ in Λ, and the smooth $L_{1}$ function is defined by:

(3) $$Smooth_{L_{1}}(z)=\left\{\begin{array}{c} \frac{z^{2}}{2},\;\;\;if\;z<1\\ |z|-0.5,\;\;otherwise. \end{array}\right.$$

### 3.2. Stereo Constrained PGD Attacks

To investigate the vulnerability of DNN-based stereo matching models, we have devised a feasible attack method grounded in the PGD approach [6]. This method maintains the photometric consistency inherent in stereo matching by adjusting the intensity of the same physical point in two images. Specifically, during adversarial training, the same perturbation is applied to the corresponding pixel pairs in the left and right images at the same time, leaving the occluded area unaffected. The image on the left serves as a reference for calculating the parallax loss, so we avoid attacking or evaluating occluded areas in the reference image. This strategy ensures that perturbations are limited to areas where parallax estimation relies on accurate matching.

Given a perturbation map $P\left(x,y\right)$, for all (x, y) in Λ, the modified intensities at each pixel location $\left(x,y\right)$ are calculated as follows:

(4) $$\begin{array}{c} I_{adv}^{L}(x,y)=I^{L}(x,y)+P(x-D(x,y),y),\\ I_{adv}^{R}(x,y)=I^{R}(x,y)+P(x,y) \end{array}$$

Let $D\left(x,y\right)$ denote the ground-truth disparity map. For two corresponding patches in the left and right images that encompass the same physical points, the absolute sum of differences between these patches will remain unchanged following the attack.

We utilize the $L_{\infty }$ norm to measure the similarity between images, ensuring that two images remain visually indistinguishable within a specified threshold.

Mathematically, for two images I and $I^{\prime }$, the L-infinity norm of their difference is defined as:

(5) $${\left\|I-I^{\prime }\right\|}_{\infty }=\underset{i}{\max}\left|I_{i}-I_{i}^{\prime }\right|$$

where $I_{i}$ and $I_{i}^{\prime }$ represent the pixel values at position i in images I and $I^{\prime }$, respectively.

To derive an $L_{\infty }$-bounded adversarial perturbation $P_{adv}$, we utilize the iterative PGD method:

(6) $$P_{t+1}^{adv}=clip_{P}^{\epsilon }\{P_{t}^{adv}+\alpha \cdot sign(\nabla_{P}L(P_{t}^{adv}))\}$$

where t=1,2,…,T and $P_{adv}^{0}$ initializes as an array of zeros. L represents the mean absolute error between the predicted disparity map for the perturbed images and the ground-truth disparity map. The function ${clip}_{\epsilon }p$ constrains the perturbation within the ϵ-ball around the corresponding zero-plane and the maximum color range. In our experiments, we set ϵ=0.06 or 0.03, α=1, and T=20.

#### 3.2.1. Attack Census Transform

The non-differentiable comparison operator in the census transform obstructs the direct backpropagation of gradients from the constructed cost volume to the input images, thereby creating a false sense of security—an issue known as the obfuscated gradient problem [41]. To ensure equitable comparisons with differentiable approaches, we replace the comparison operator with a combination of subtraction and the sigmoid function, providing a smooth and differentiable alternative.

(7) $$a>b\approx sigmoid\left(a-b\right)\cdot C$$

We utilize a large constant (i.e., $C=10^{5}$) to drive the output of the sigmoid function towards binary states, ensuring values are close to either zero or one. In the absence of this differentiable approximation, our method—devoid of the contextual feature backbone—would become impervious to adversarial attacks, as the gradient pathways would be entirely obstructed.

#### 3.2.2. Robustness of Census Transform

From an adversarial perspective, the binary patterns generated by the census transform present a significant challenge to manipulation due to the inherent robustness of the comparison operator. Given a fixed perturbation threshold for pixel differences, neighboring pixels will remain unaffected if their difference from the central pixel exceeds twice this threshold. The difficulty in altering the cost between corresponding binary patches is further compounded when photometric consistency is maintained during attacks. Specifically, if a neighboring pixel is present in both the left and right binary patches, its relative magnitude to the central pixel remains consistent across both patches, irrespective of intensity variations. This study seeks to explore whether this highly non-linear operator can effectively safeguard deep neural networks (DNNs) against adversarial attacks.

## 4. Experiments and Discussion

This section begins with a detailed description of the training and testing process of the proposed method. Then, the results of Sim2Real’s cross-domain generalization are introduced, and finally the adversarial robustness of the method is evaluated.

### 4.1. Settings and Experimental Details

#### 4.1.1. Data

To validate our methodology, we employed the public datasets SceneFlow [18] and KITTI 2015 [19] as well as proprietary data we collected. The SceneFlow dataset is a comprehensive synthetic corpus featuring 35,454 training images and 4370 test images at a resolution of 540 × 960. It is extensively utilized for pretraining DNN-based stereo matching techniques due to its provision of dense ground-truth disparity. The KITTI 2015 dataset, capturing real-world driving scenarios, comprises 200 training and 200 test images at a resolution of 375 × 1242. Here, depth information is derived from LiDAR, resulting in sparser disparity data. Additionally, we evaluated the pretrained model at a quarter-resolution on the KITTI 2012 [42] and Middlebury [43] datasets, further demonstrating the model’s adaptability across varied environments and resolutions.

**Figure A1:**
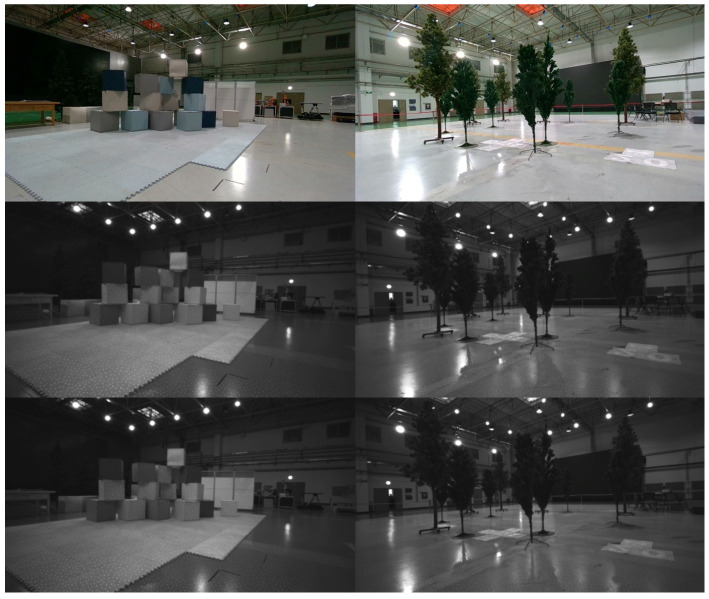
The sample of real infrared binocular data captured for training the model.

**Figure A2:**
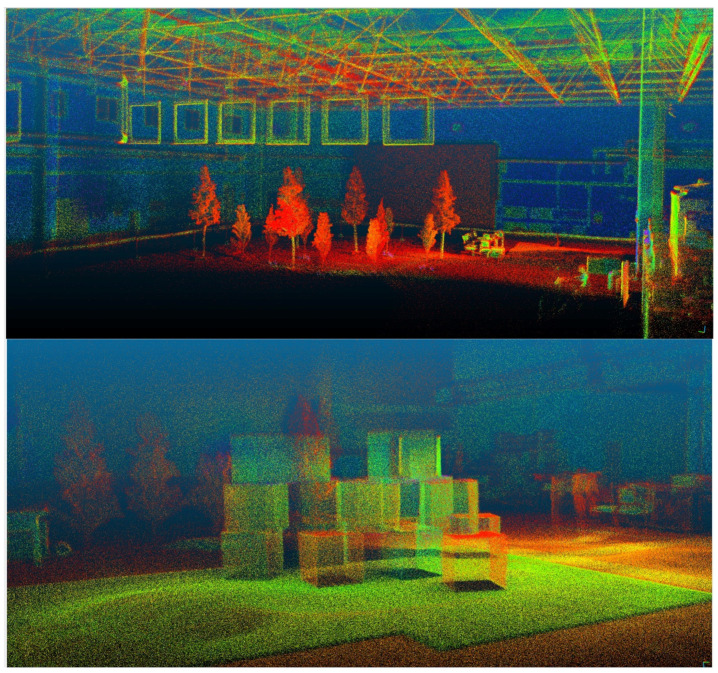
Laser depth map for dataset annotation.

**Figure A3:**
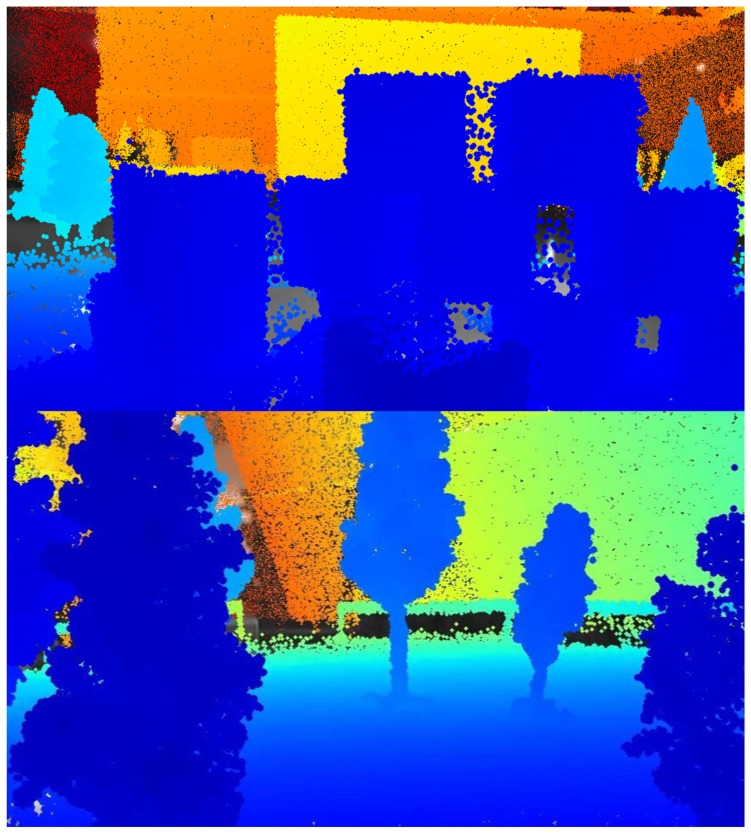
The sample of annotated infrared stereo dataset (ground truth).

The datasets capturing real obstacles such as boxes and trees by an infrared binocular camera are shown in Figure A1. To obtain the label of the depth of the pixels, laser LiDAR is used to obtain the depth map shown in Figure A2. And the sample of annotation results is as shown in Figure A3.

#### 4.1.2. Experimental Details

- Training details

Our method is implemented in PyTorch 1.9.0 and trained end-to-end using the Adam optimizer with $\beta_{1}=0.9$ and $\beta_{2}=0.999$. Prior to training, whole images undergo color normalization to adapt to the infrared images, which are consistently in grayscale. We use a batch size of eight, distributed across two Nvidia 3090 GPUs, with random 240 × 576 crops from the input images. The maximum disparity level is set at 192, with values beyond the threshold disregarded while training.

For the SceneFlow dataset, we train our model from scratch for 20 epochs, maintaining a constant learning rate of 0.001. When transitioning to the KITTI 2015 dataset, we divide the 200 images into a training set of 140 and a validation set of 60. For our own dataset, we split 420 images into a training set of 320 and a validation set of 100. The model, pretrained on SceneFlow, is then fine-tuned for an additional 600 epochs, with the best model selected based on validation performance.

**Figure 2:**
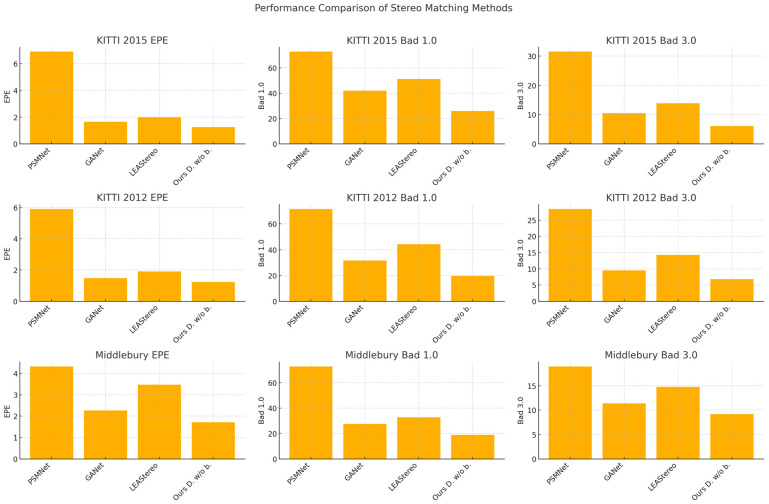
Comparisons of performance on standard datasets.

Scenarios where the feature backbone for extracting contextual information from the left image is omitted are shown in Figure 2. This model is referred to as “ours Double w/o backbone” or “ours D. w/o b.” in the following text.

- Evaluation Metrics

We adopt the provided protocols with two datasets, employing three key metrics for assessment [44,45]: end-point error (EPE) in pixels, Bad 1.0 [%], and Bad 3.0 [%]. EPE measures the disparity end-point error in pixels, while Bad 1.0 [%] and Bad 3.0 [%] indicate the percentage of pixels with disparity errors exceeding one pixel and three pixels, respectively.

- Baseline methods

We evaluate our approach against leading deep stereo matching methods: PSMNet [22], GANet [27], and LEAStereo [29]. For these comparisons, we utilize their publicly available code and pretrained model checkpoints.

### 4.2. Results and Discussion

In this part, the primary focus is on the generalization, robustness, and adversarial attack resilience of the algorithm. Initially, the performance of our algorithm, along with comparison algorithms, is evaluated across multiple standard datasets. Subsequently, we qualitatively analyze the superiority of algorithm on the KITTI 2015 dataset and real-world infrared binocular images through the adversarial patch attack maps and depth prediction maps.

#### 4.2.1. Sim2Real Cross-Domain Generalizability

To test the hypothesis that decoupling the cost computation from the dataset-specific feature backbone enhances cross-domain generalization in stereo matching, we employed the KITTI and Middlebury training datasets to assess models initially trained on the SceneFlow dataset [43]. As depicted in Figure 2, we examined the cross-domain generalization capabilities of the model by evaluating it on the KITTI 2015, KITTI 2012, and Middlebury datasets without any fine-tuning. This direct comparison not only underscores the effectiveness of our approach in bridging the gap between synthetic and real-world data but also demonstrates its substantial superiority over previous methodologies.

The experimental outcomes reveal that our design, which integrates a non-parametric cost constructed through multi-scale census transformation with a generalized cost aggregation/optimization framework via deep neural networks, achieves markedly higher cross-domain consistency than the baseline models.

#### 4.2.2. Adversarial Patch Attack

**Figure 3:**
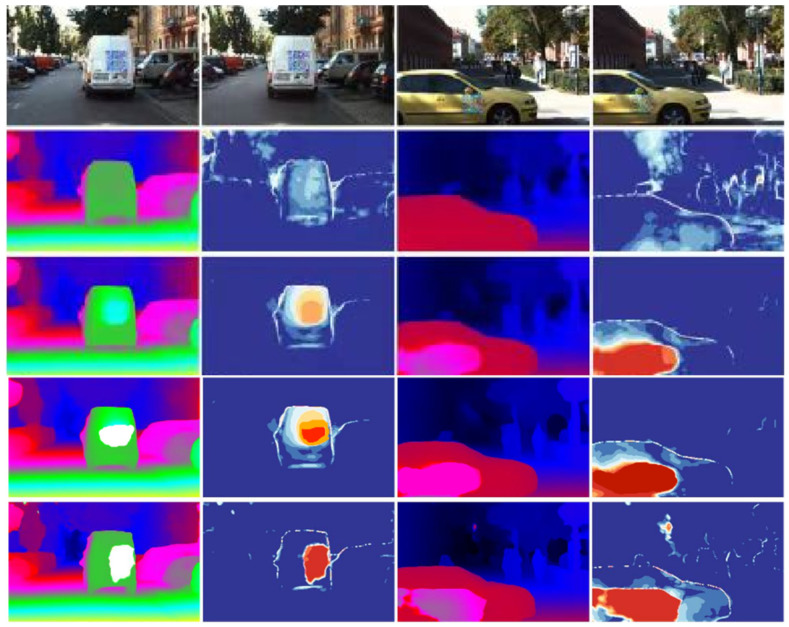
Illustration of the adversarial patch attack. The first row is the image selected in the KITTI 2015 with ground truth but attacked by PGD method. The second row to the last are the results of: our method, LEAStereo, PSMNet, and GANet, where the first and third lines are the depth maps and the second and fourth lines are the error maps generated by the result in the first and third lines compared with the ground truth. The colors in the depth maps (in first and third lines) given by our methods are light to dark in the following order: white, green, pink, red, blue, and dark which means from near to far.

To evaluate the potential for adversarial vulnerabilities in realistic applications such as autonomous driving, we selected two scenarios wherein adversarial patches can be adhered to flat surfaces. We simulated the application of these patches by aligning their ground-level parallax with corresponding regions in the original stereo images. For each image pair, we executed 1000 iterations of stereo constrained projected gradient descent (PGD) attacks, with the outcomes illustrated in Figure 3. The initial row in Figure 3 depicts a confrontational plaque affixed to a planar surface within a binocular scene. Subsequent rows showcase the results from various stereo matching algorithms—our method, LEAStereo, PSMNet, and GANet. The error mappings in the right column of each row, where red and blue indicate high and low errors, respectively, visually underscore the differential impacts of the attacks.

**Figure A4:**
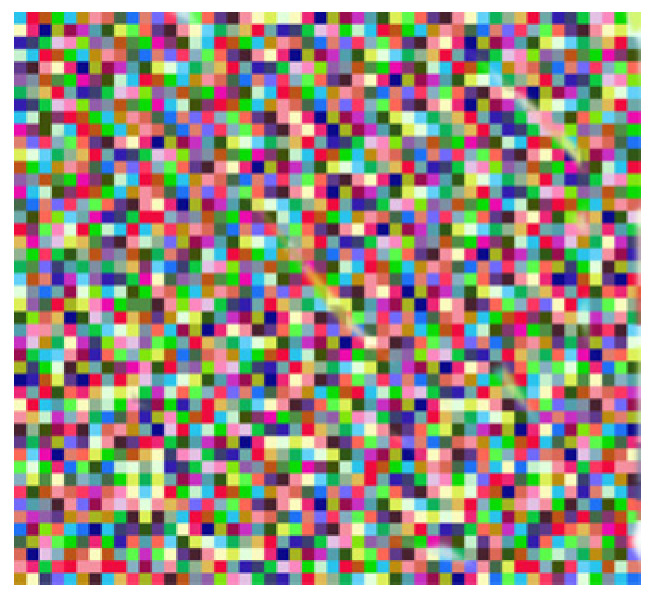
The PGD attack attached (the pixels are generated randomly).

According to Figure 3, we observe a significant change in the parallax map of PSMNet and GANet, which results in the affected surfaces appearing closer together than they were attacked. In contrast, LEAStereo exhibits considerably fewer errors, as indicated by the yellow and orange regions. Our method, on the other hand, produces a depth map that remains unaffected by adversarial patch attacks. The error maps further illustrate that our approach yields more accurate results and less errors, demonstrating its robustness against such attacks. The close-up image for the attached attack is in Figure A4.

#### 4.2.3. Result of KITTI 2015

**Figure 4:**
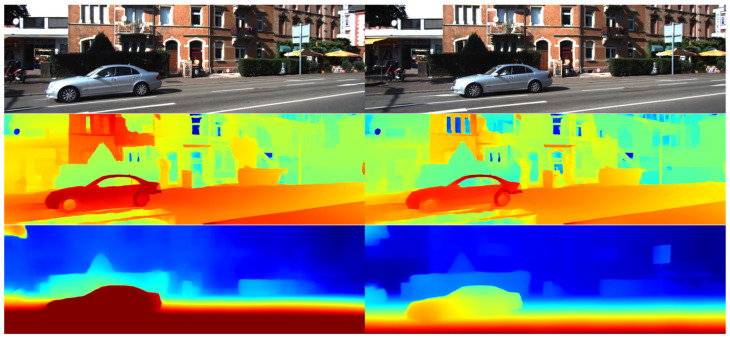
Depth prediction map given by baseline and our D. w/o b. method.

Figure 1 demonstrates the capability of the proposed stereo matching method to concurrently estimate left and right parallax with satisfactory accuracy. Utilizing a pretrained model from the SceneFlow dataset, we further enhanced its performance by fine-tuning on the left view of the KITTI 2015 dataset over an additional 50 epochs. Despite the absence of differential data for the right view in KITTI 2015, our neural networks have adeptly learned to construct dense volumes that effectively generate accurate right parallax maps, as illustrated in Figure 4.

The first row displays the color images of the left and right views from the KITTI 2015 dataset. The second row presents the results from GANet and PSMNet. The third row displays the outputs from LEAStereo and our proposed method. Depth prediction images are based on the left view, as disparity data are available for the left eye only.

As evident from the images, without the benefit of stereo matching using both views, GANet and PSMNet exhibit severe issues in depth prediction, while LEAStereo suffers from partial object disappearance. In contrast, our method, which integrates information from the right view, produces clear depth predictions, with the contours of the motorcycle on the left and the signpost on the right distinctly visible.

#### 4.2.4. Result of Real Infrared Binocular Stereo Matching

In our experiments, we observed that stereo matching algorithms are highly sensitive to environmental lighting conditions. Both strong and weak light can cause stereo matching to fail, resulting in incorrect depth maps. To further investigate the algorithm’s applicability in real-world scenarios, we conducted comparative experiments under various lighting conditions. We found that in low-light environments, small objects such as branches tend to be lost, while in strong light, large obstacles such as boxes are not significantly affected. Therefore, it is reasonable to conduct stereo matching experiments on small objects in strong light and on large obstacles in low light to draw meaningful comparisons.

**Figure 5:**
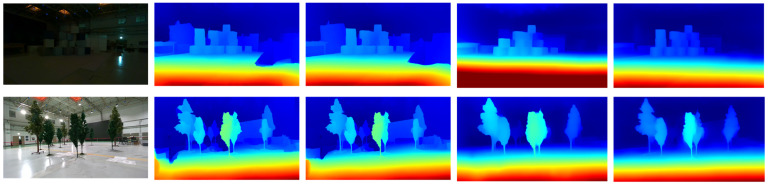
The result of depth prediction in real complex environment by infrared camera.

As shown in Figure 5, the results illustrate the performance under different lighting conditions. The first row presents the outcomes in a low-light environment, with the leftmost image depicting the original condition. The subsequent images from left to right display the results of the baseline methods: GANet, PSMNet, LEAStereo, and our method D. w/o b. The second row follows the same order but under high-light conditions.

The baseline methods were tested using images with the infrared texture removed, as these methods were not originally designed for infrared binocular stereo matching. In contrast, our method utilizes infrared binocular images, as illustrated in Figure A1. This adaptability highlights the robustness of our algorithm, demonstrating its capability to adaptively function effectively with various types of cameras.

The results are readily apparent: both GANet and PSMNet produce erroneous depth predictions on the ground under varying lighting conditions, whether in bright or dark environments. LEAStereo performs somewhat better but still fails to detect certain objects. In contrast, our method is the only one capable of accurately identifying the tree behind the boxes in the first row and the black screen in the second row. These findings not only highlight the accuracy of our model but also underscore its robustness and generalization capabilities in complex real-world scenarios.

## 5. Conclusions

In conclusion, we present a novel stereo matching framework that synergistically integrates classical multi-scale census transformations with end-to-end trainable deep neural networks. This approach significantly enhances robustness and generalizability in infrared binocular stereo matching by leveraging handcrafted features to mitigate the adversarial vulnerabilities inherent in DNN-based models. We address the susceptibility of conventional stereo matching networks to physically realizable adversarial attacks that manipulate stereo constraints during perturbation learning. By revising the DNN architecture traditionally employed in cost volume calculations—specifically by excluding it from the matching phase—we achieve marked improvements in adversarial robustness and simulation-to-reality (Sim2Real) generalization on the SceneFlow and KITTI 2015 datasets without the need for fine-tuning. Furthermore, our method facilitates more precise and reliable depth estimation in complex, real-world infrared imagery.

Despite these advancements, our approach has certain limitations. The reliance on handcrafted features like the census transform may not fully capture the rich semantic information available in images, potentially limiting performance in scenarios with minimal texture or repetitive patterns. Additionally, while our method enhances robustness against specific adversarial attacks, it may not address all forms of adversarial strategies, particularly those exploiting other network vulnerabilities.

Future research directions include exploring hybrid models that combine handcrafted features with learned representations to further enhance performance and robustness. Investigating adaptive mechanisms that dynamically balance robustness and accuracy based on the operating environment could also prove beneficial. Moreover, extending our framework to accommodate other sensing modalities and evaluating its efficacy under diverse real-world conditions will be essential steps toward deploying reliable stereo matching systems in safety-critical applications like autonomous navigation.

## Data Availability

The datasets presented in this article are not readily available because of related patent applications and subsequent research papers. Requests to access the datasets should be directed to the corresponding author.
